# Have we gotten better at diagnosing PDAC before it is too late?: Temporal trends in stage at diagnosis of pancreatic ductal adenocarcinoma in the United States

**DOI:** 10.1007/s10552-026-02266-6

**Published:** 2026-10-07

**Authors:** Dina Hauptschein, Paul Kang, Surabhi Amar

**Affiliations:** Creighton University Arizona Health Education Alliance, 3100 N. Central Ave., Phoenix, AZ 85012 USA

**Keywords:** Pancreatic ductal adenocarcinoma (PDAC), Surveillance, Epidemiology, and End Results (SEER), Early detection, Incidence trends, Localized disease

## Abstract

**Purpose:**

Pancreatic ductal adenocarcinoma (PDAC) remains one of the deadliest cancers in the United States, with most patients not being diagnosed until they present with symptomatic, advanced-stage disease. National trends in stage-specific PDAC incidence were analyzed to assess trends in stage-at-diagnosis over time and to evaluate whether advances in diagnosis and high-risk screening programs have helped shift detection towards earlier stages.

**Methods:**

A retrospective cohort study was conducted using the Surveillance, Epidemiology, and End Results (SEER) database. Patients diagnosed with PDAC between the years 2004 and 2023 were included. Age-adjusted incidence rates were calculated per 100,000 people. Trends were assessed using Joinpoint regression to estimate the average annual percent change (AAPC). Multivariable Negative Binomial regression was used to estimate incidence rate ratios (IRRs) with stage-specific cases, adjusting for age, sex, race/ethnicity, and calendar year.

**Results:**

Between the years 2004 and 2023, age-adjusted incidence rates of localized, regional, and distant-stage PDAC were 1.04, 1.26, and 4.94 per 100,000 population, respectively. Localized PDAC demonstrated the greatest increase over time, with an AAPC of 6.00% (95% CI: 4.96–7.49; p < 0.001), while distant-stage disease exhibited a small increase over time (AAPC 1.11%; 95% CI: 1.02–1.21; p < 0.001) and regional-stage disease did not change significantly (AAPC 0.27%; 95% CI: −1.18 to 1.92; p = 0.62). Localized-stage incidence accelerated further after approximately 2015, with APC increasing to 14.7% between 2015 and 2019. Similar localized-stage increases were observed across sex and racial/ethnic subgroups. In multivariable Negative Binomial regression, increasing age and Non-Hispanic Black race were associated with higher PDAC incidence, while female sex was associated with lower regional and distant incidence rates.

**Conclusions:**

The findings suggest a stage shift toward increased diagnosis of localized PDAC over time. These trends may reflect improvements in imaging, surveillance of high-risk individuals, advances in endoscopic detection, and increasing diagnostic intensity, although rising underlying PDAC burden may also contribute. Further prospective studies are needed to determine whether these trends ultimately translate into meaningful improvements in survival outcomes.

**Supplementary Information:**

The online version contains supplementary material available at https://doi.org/10.1007/s10552-026-02266-6.

## Introduction

Pancreatic ductal adenocarcinoma (PDAC) is the 10th most diagnosed cancer in 2025 according to the National Cancer Institute, but accounts for the third largest share of cancer-related deaths [1]. Despite modest improvements in survival over time, the reported 5-year survival for PDAC remains between 10 and 14% [2].

One of the most reliable predictors of outcome for PDAC is the stage at diagnosis. Patients diagnosed with localized disease show significantly better survival outcomes. However, this group represents a vast minority, with most patients being diagnosed with either regional or metastatic disease. Singhi et al. note that this likely reflects both the lack of an effective screening strategy for the general population and the nonspecific setting in which early PDAC typically presents [4]. Poor outcomes are largely driven by the high proportion of patients who present with advanced-stage disease at diagnosis [3].

There have been several advancements over the past couple of decades that have improved the ability to diagnose PDAC at earlier stages. Imaging is better than it used to be, high-risk surveillance is more widely used, and endoscopic ultrasound has become more common in clinical practice. There has also been growing interest in biomarker-based detection strategies. However, there has been no definitive research confirming whether these changes have actually translated into earlier diagnosis at the population level [5–7].

Pancreatic cancer incidence and mortality continue to rise globally and are projected to become an even greater public health risk over the next several decades. Rahib et al. project that pancreatic cancer may become the second-leading cause of cancer-related death in the United States by 2030, reflecting demographic shifts as well as rising incidence rates [8]. Several factors have been implicated in these trends, including aging populations, the increasing prevalence of obesity and diabetes, and improved diagnostic detection, as described by Klein [9]. According to the CDC’s Behavioral Risk Factor Surveillance System, from 2011 to 2023, the percentage of adults in the US with obesity and diabetes increased by 23% and 21%, respectively [10]. Understanding whether the increased diagnosis of localized PDAC reflects true stage migration, a rising disease burden, or both has important implications for screening and surveillance strategies.

Most of the existing literature has focused on incidence and overall survival. Comparatively less attention has been given to how the stage at diagnosis may have shifted over time, particularly in large, population-based datasets. Therefore, understanding whether a stage migration toward localized disease is occurring has meaningful implications for early detection strategies and future screening strategies.

In this study, we used the Surveillance, Epidemiology, and End Results (SEER) database to evaluate temporal trends in PDAC stage at diagnosis from 2004 to 2023.

## Methods

Age-adjusted incidence rates were derived from the Surveillance, Epidemiology, and End Results (SEER) “Rate-Sessions” Program using the SEER*Stat software (version 8.4.4; Surveillance Research Program, National Cancer Institute) [11]. Rates were calculated from the SEER research data covering 2000 through 2023 and drawn from 21 registries across the United States [12]. PDAC cases were identified using the SEER Recode 2020 Revision code 9.3.9.2, as defined by the AYA Site Recode 2020 Revision. The dataset included demographic variables—age (per 5-year increments), sex (male and female), and race/ethnicity (Non-Hispanic White, Non-Hispanic Black, Non-Hispanic Asian or Pacific Islander, Non-Hispanic American Indian/Alaska Native, and Hispanic of all races), as well as stage at diagnosis (in situ, localized, regional, and distant) as a clinical variable. Cases with missing or unknown race/ethnicity, unknown age categories, or staging information were excluded from all analyses. No upper or lower age restriction was applied; all cases with a known age at diagnosis were retained. Data from 2004 onward were used to ensure completeness and consistency across registry coverage. Of the records screened, 18,432 rows were removed for unknown age, diagnosis year before 2004, or unknown race/ethnicity. A further 20,000 rows were removed for unknown stage at diagnosis, and 12,000 rows that repeated the same population denominator across stage categories were removed so that each population stratum was counted only once. The rows excluded for unknown stage correspond to 11,234 PDAC cases (4.7% of the 238,645 cases identified), yielding a final cohort of 227,411 PDAC cases (Figure S1). Counts described as rows in Figure S1 refer to cells of the aggregated SEER*Stat rate session rather than to individual patients; the final grid comprises 4,000 population cells (20 age groups × 2 sexes × 5 race/ethnicity categories × 20 years of diagnosis).

Age-adjusted incidence rates per 100,000 population were calculated stratified by sex, race/ethnicity, and stage at diagnosis. The Average Annual Percent Change (AAPC) was calculated to assess trends in age-adjusted rates across the entire study period, as well as stratified by sex, race/ethnicity, and stage at diagnosis. Additionally, temporal trends in age-adjusted incidence rates were evaluated using Joinpoint regression, implemented in the Joinpoint Regression Program [13], which fits piecewise log-linear models to identify statistically significant inflection points in trend and to estimate annual percent changes (APCs). AAPCs and APCs were estimated across strata defined by sex, race/ethnicity, and stage at diagnosis; additional stratified analyses were conducted to evaluate whether temporal trends within sex and stage subgroups varied by racial/ethnic group. Multivariable Negative Binomial regression using the “MASS” package was performed to estimate incidence rate ratios (IRRs) for stage-specific PDAC cases in relation to demographic factors. In these models the outcome was the stage-specific PDAC case count within strata defined by age group, sex, race/ethnicity, and year of diagnosis, with the natural logarithm of the corresponding population person-years included as an offset so that the coefficients estimate incidence rate ratios; age was entered per 5-year increment, derived from the SEER age recode with <1 year olds and 90+ (20 categories). Overdispersion was assessed using the “performance” package. P-values were two-sided and p<0.05 was considered statistically significant. All analyses were conducted within R Studio version 2026.08.1+Build 195 (POSIT, PBC, Boston, MA). Piecewise (non-linear) APC estimates for each segment identified by Joinpoint are reported in Supplementary Tables S1 and S2. All-stage trends stratified by sex and by race/ethnicity are shown in Supplementary Figures S2 and S3, and the percent distribution of stage at diagnosis across the study period is shown in Supplementary Figure S4.

## Results

Between 2004 and 2023, age-adjusted incidence rates of pancreatic ductal adenocarcinoma (PDAC) differed meaningfully by sex, race/ethnicity, and stage at diagnosis (Table 1). The final analytic cohort comprised 227,411 PDAC cases, with an overall age-adjusted incidence rate of 7.25 per 100,000 population (95% CI: 7.22–7.28). Males demonstrated a higher overall age-adjusted incidence rate than females (8.31 vs. 6.35 per 100,000 population). Across racial/ethnic groups, Non-Hispanic Black individuals exhibited the highest incidence rate (8.91 per 100,000), followed by Non-Hispanic White (7.37), Hispanic (6.53), Non-Hispanic American Indian/Alaska Native (5.56), and Non-Hispanic Asian or Pacific Islander individuals (NHAPI) (5.38). By stage, incidence rates increased markedly with advancing disease: localized PDAC had an age-adjusted rate of 1.04 per 100,000, regional disease 1.26 per 100,000, and distant-stage disease 4.94 per 100,000; no in situ cases were identified in the analytic cohort.

**Table 1:** Case counts, population denominators, and age-adjusted incidence rates of pancreatic ductal adenocarcinoma, overall and by sex, race/ethnicity, and stage at diagnosis, SEER 21 registries, 2004–2023

Variable	PDAC cases N (2004–2023)	Population denominator N (2004–2023)	Age-adjusted rate per 100,000 (95% CI)
Overall	227,411	2,896,790,700	7.25 (7.22, 7.28)
Sex			
Male	118,863	1,431,778,853	8.31 (8.27, 8.36)
Female	108,548	1,465,011,847	6.35 (6.31, 6.39)
Race/ethnicity			
Non-hispanic white	156,252	1,547,819,562	7.37 (7.34, 7.41)
Non-hispanic black	28,100	352,993,768	8.91 (8.81, 9.02)
Non-hispanic Asian/Pacific Islander	13,163	252,275,647	5.38 (5.29, 5.47)
Non-hispanic AI/AN	956	18,670,530	5.56 (5.20, 5.94)
Hispanic	28,940	725,031,193	6.53 (6.45, 6.60)
Stage at diagnosis			
In situ	0	2,896,790,700	–
Localized	31,743	2,896,790,700	1.04 (1.03, 1.05)
Regional	40,040	2,896,790,700	1.26 (1.25, 1.28)
Distant	155,628	2,896,790,700	4.94 (4.92, 4.97)

*AI/AN* American Indian/Alaska Native, *CI* confidence interval

Rates are per 100,000 population, age-adjusted to the 2000 US standard population. Denominators are person-years at risk over the study period. The overall and stage-specific rates share the total denominator of 2,896,790,700 person-years, since the whole population is at risk of each stage; the sex and race/ethnicity denominators each sum to this total

**Fig. 1:**
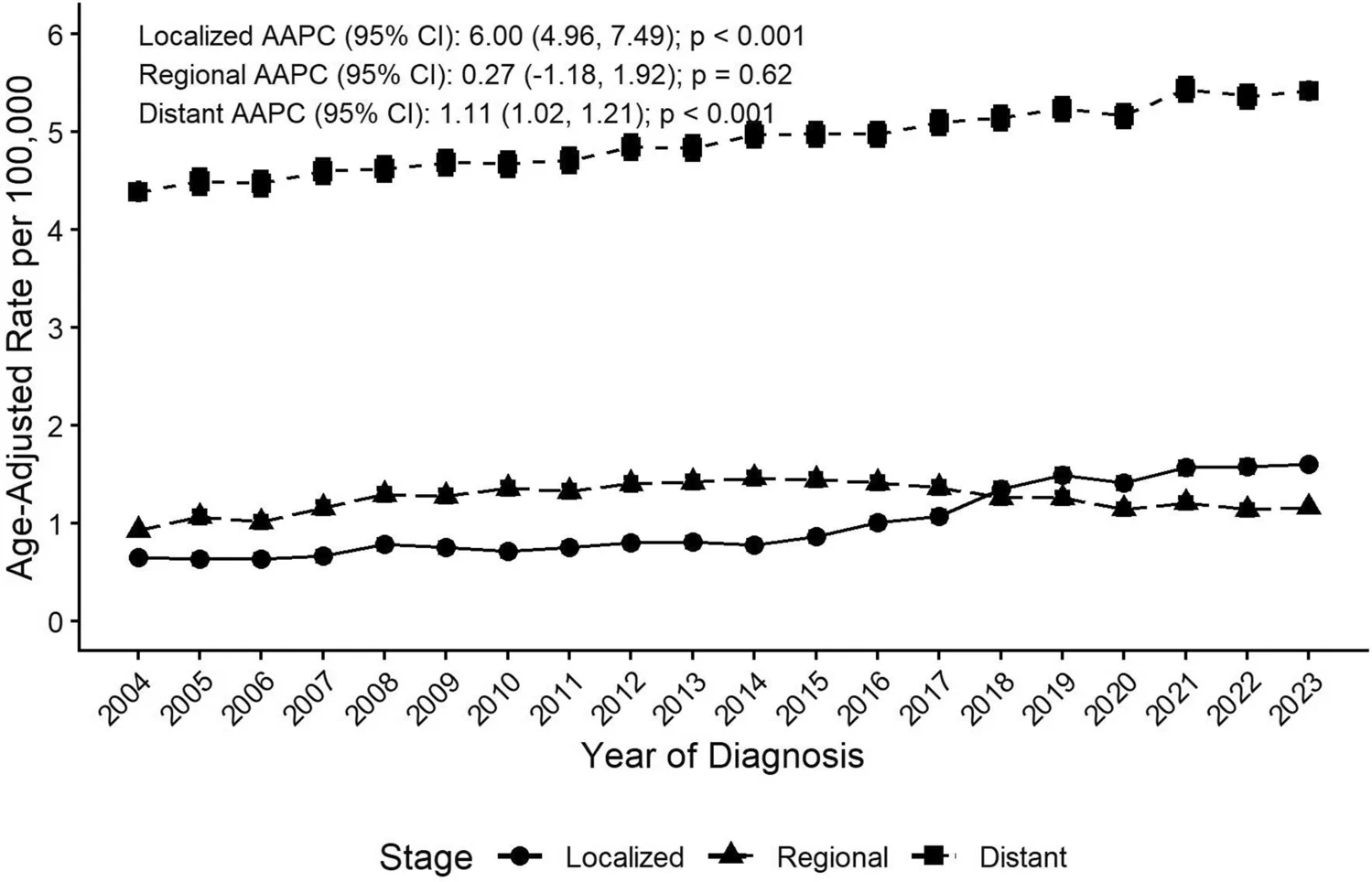
Average annual percent change in age-adjusted incidence rates of pancreatic ductal adenocarcinoma, by stage at diagnosis, 2004–2023

**Fig. 2:**
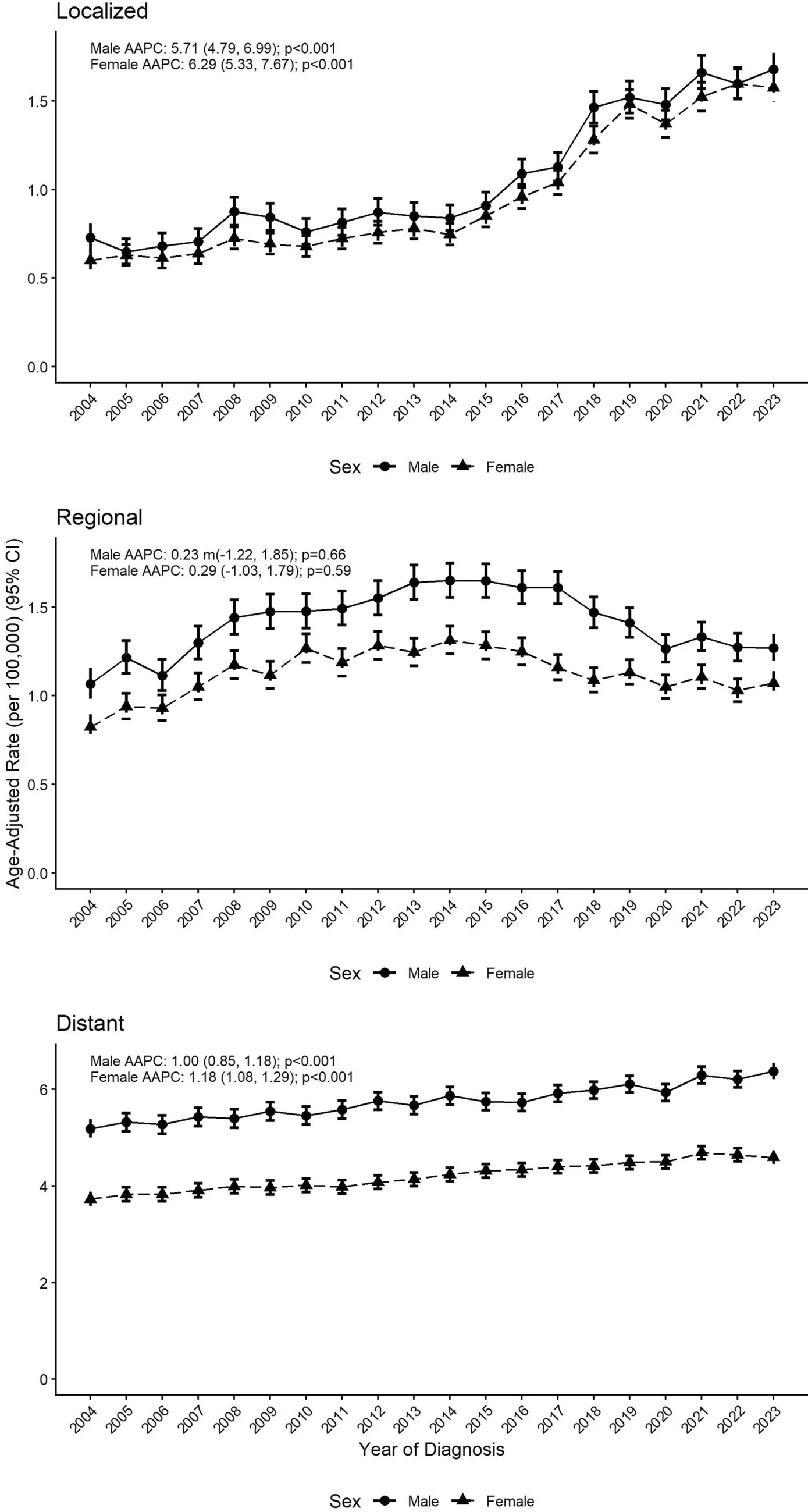
Average annual percent change in age-adjusted incidence rates of pancreatic ductal adenocarcinoma, by stage at diagnosis and sex, 2004–2023

However, the incidence of PDAC appears to have increased over time within the localized staging (AAPC of 6.00%; 95% CI: 4.96–7.49; p < 0.001), while distant staging exhibited a small increase in PDAC incidence over time (AAPC of 1.11%; 95% CI: 1.02–1.21; p < 0.001). Regional-stage incidence did not change significantly across the study period (AAPC of 0.27%; 95% CI: −1.18 to 1.92; p = 0.62) (Fig. 1). Figure 2 presents sex-stratified age-adjusted PDAC incidence trends by stage from 2004 to 2023. Localized-stage incidence increased significantly and comparably for both males (AAPC: 5.71%; 95% CI: 4.79–6.99; p < 0.001) and females (AAPC: 6.29%; 5.33–7.67; p < 0.001). Regional-stage incidence did not change significantly in either sex over the full study period. Males showed a non-significant overall change (AAPC: 0.23%; −1.22 to 1.85; p = 0.66), with rates rising until approximately 2014 and declining thereafter through 2023. Females showed a similar pattern (AAPC: 0.29%; −1.03 to 1.79; p = 0.59), with an early rise followed by a non-significant decline after 2014 (Supplementary Table S2). Distant-stage incidence increased slowly but significantly in both males (AAPC: 1.00%; 0.85–1.18; p < 0.001) and females (AAPC: 1.18%; 1.08–1.29; p < 0.001).

**Fig. 3:**
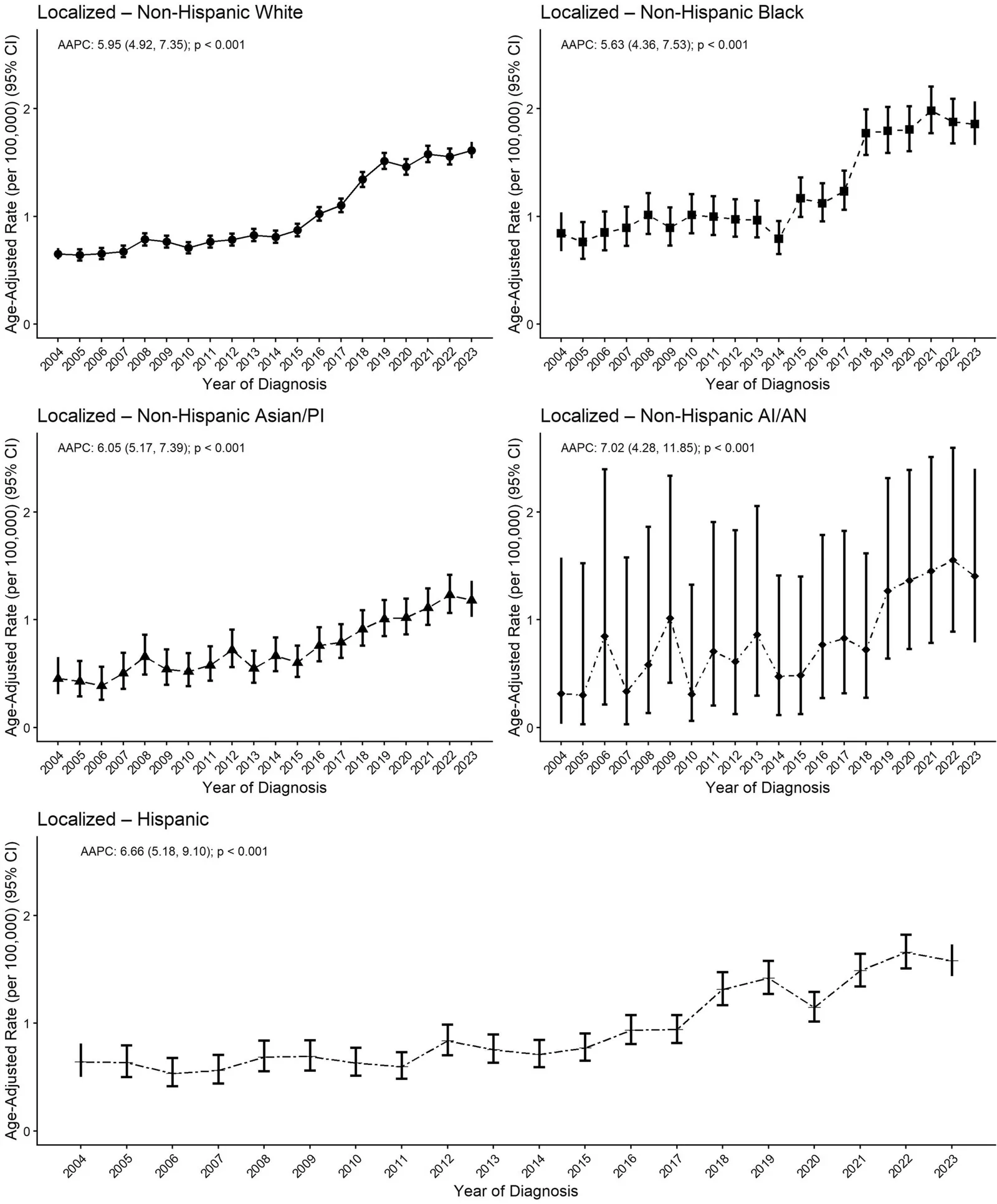
Average annual percent change in age-adjusted incidence rates of localized-stage pancreatic ductal adenocarcinoma, by race/ethnicity, 2004–2023

After stratification of localized diagnosis by race/ethnicity, Non-Hispanic White individuals demonstrated a sharp post-2015 acceleration (AAPC: 5.95%; 95% CI: 4.92–7.35; p < 0.001), with accelerated increases from 2015 to 2019 (piecewise APC: 14.8%; 95% CI: 11.2–19.8; p < 0.001). Non-Hispanic Black individuals showed a similarly steep increase (AAPC: 5.63%; 4.36–7.53; p < 0.001), with similar increases of 14.1% per year from 2014 to 2019. Hispanic individuals also had a significant overall AAPC (6.66%; 5.18–9.10; p < 0.001), with rates accelerating to about 9% per year after 2014. Non-Hispanic Asian or Pacific Islander individuals also showed a significant and sustained increase (AAPC: 6.05%; 5.17–7.39; p < 0.001). Non-Hispanic American Indian/Alaska Native individuals demonstrated the largest variability, reflecting a smaller population size, yet still showed a significant overall upward trend (AAPC: 7.02%; 4.28–11.85; p < 0.001) (Fig. 3). Piecewise estimates for each racial/ethnic group are reported in Supplementary Table S2.

**Fig. 4:**
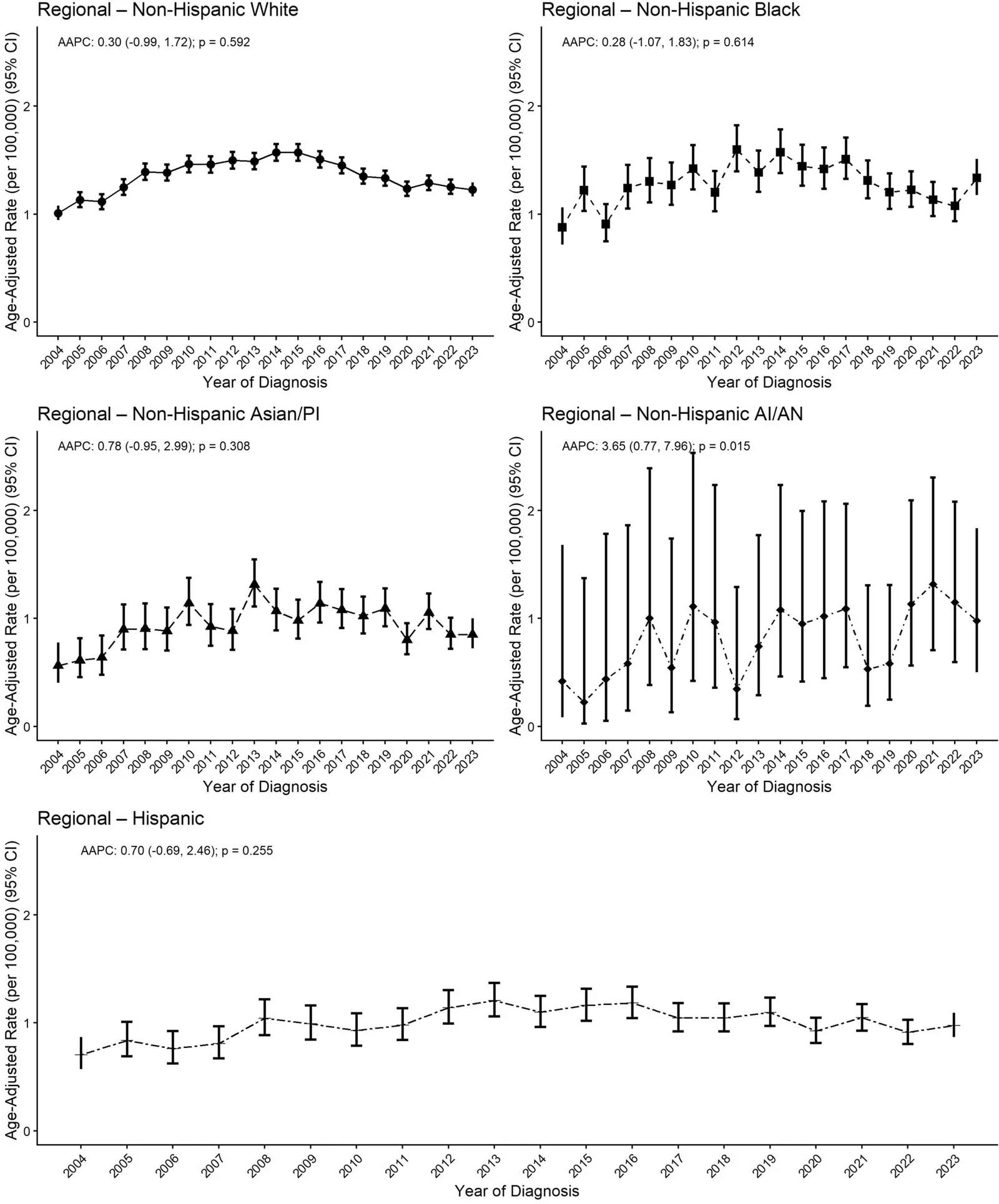
Average annual percent change in age-adjusted incidence rates of regional-stage pancreatic ductal adenocarcinoma, by race/ethnicity, 2004–2023

**Fig. 5:**
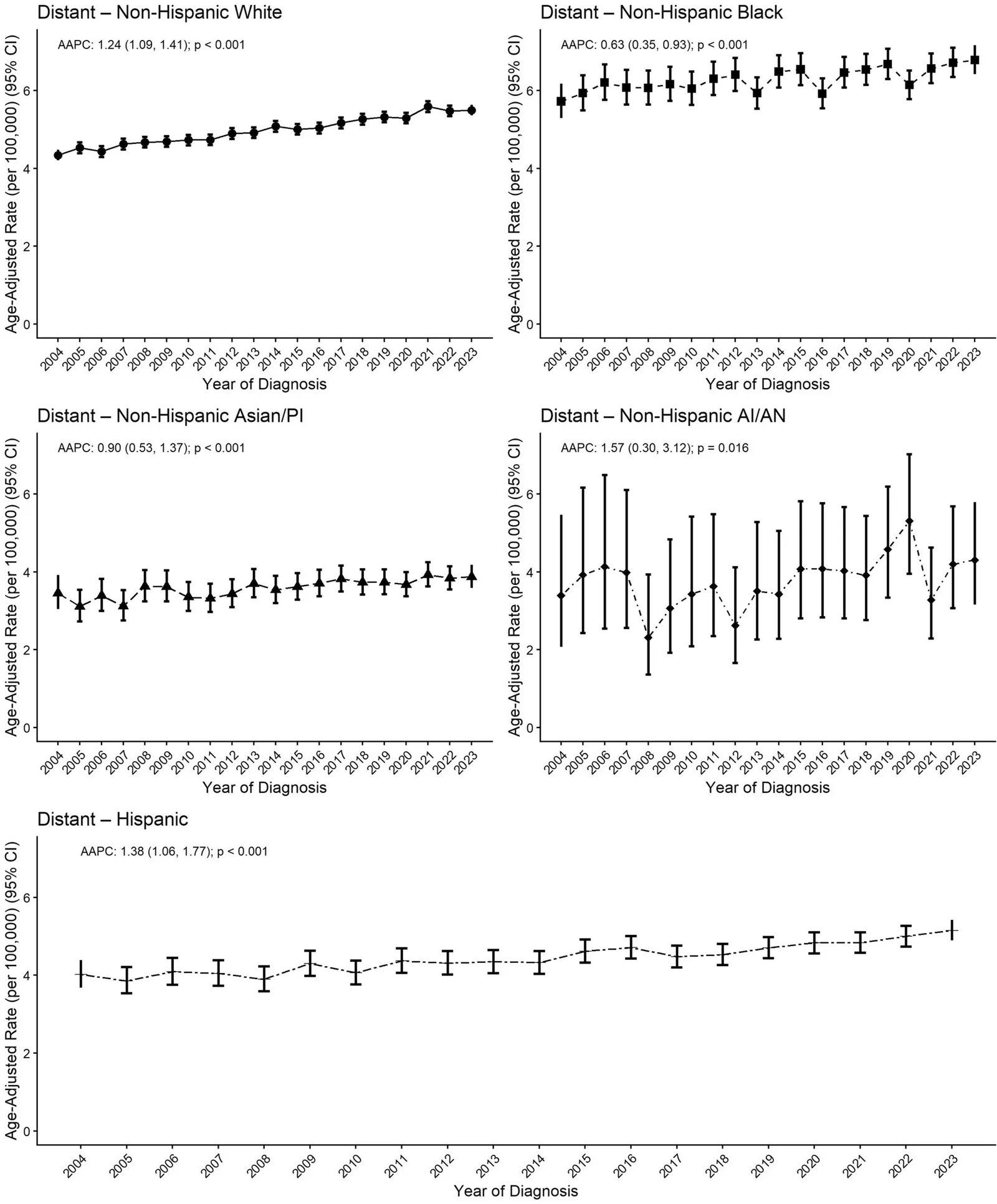
Average annual percent change in age-adjusted incidence rates of distant-stage pancreatic ductal adenocarcinoma, by race/ethnicity, 2004–2023

Regional-stage trends by race/ethnicity were flat or declining in most groups (Fig. 4). No significant change was observed among Non-Hispanic White (AAPC: 0.30%; 95% CI: −0.99 to 1.72; p = 0.59), Non-Hispanic Black (AAPC: 0.28%; −1.07 to 1.83; p = 0.61), Non-Hispanic Asian or Pacific Islander (AAPC: 0.78%; −0.95 to 2.99; p = 0.31), or Hispanic individuals (AAPC: 0.70%; −0.69 to 2.46; p = 0.26); only Non-Hispanic American Indian/Alaska Native individuals showed a significant regional-stage increase (AAPC: 3.65%; 0.77–7.96; p = 0.015). Distant-stage incidence increased modestly but significantly in all five groups (Fig. 5): Non-Hispanic White (AAPC: 1.24%; 1.09–1.41; p < 0.001), Non-Hispanic Black (AAPC: 0.63%; 0.35–0.93; p < 0.001), Non-Hispanic Asian or Pacific Islander (AAPC: 0.90%; 0.53–1.37; p < 0.001), Non-Hispanic American Indian/Alaska Native (AAPC: 1.57%; 0.30–3.12; p = 0.016), and Hispanic individuals (AAPC: 1.38%; 1.06–1.77; p < 0.001).

In stage-specific multivariable Negative Binomial regression models, including age, sex, race, and year of diagnosis, the associations between these covariates and PDAC incidence varied by stage at diagnosis (Table 2). Age, per 5-year increase, was associated with a 52% higher rate for localized disease (IRR: 1.52; 95% CI: 1.51–1.53; p < 0.001), a 59% higher rate for regional disease (IRR: 1.59; 1.57–1.60; p < 0.001), and a 68% higher rate for distant disease (IRR: 1.68; 1.67–1.69; p < 0.001). The association between sex and incidence differed in direction by stage. Compared with the male sex, female sex was associated with a modestly higher rate of localized disease (IRR: 1.06; 1.01–1.11; p = 0.020) but lower rates of regional (IRR: 0.81; 0.75–0.88; p < 0.001) and distant disease (IRR: 0.73; 0.69–0.78; p < 0.001). Relative to Non-Hispanic White individuals, Non-Hispanic Black individuals had significantly higher rates of localized (IRR: 1.31; 1.23–1.40; p < 0.001) and distant disease (IRR: 1.43; 1.30–1.56; p < 0.001), with a non-significant association for regional disease (IRR: 1.10; 0.99–1.22; p = 0.083). Non-Hispanic Asian or Pacific Islander individuals had significantly lower rates across all stages. Non-Hispanic American Indian/Alaska Native individuals had a significantly lower rate of regional disease (IRR: 0.74; 0.61–0.90; p = 0.003), with non-significant differences for localized (IRR: 0.90; 0.75–1.07; p = 0.22) and distant disease (IRR: 0.91; 0.80–1.03; p = 0.15). Hispanic individuals had a significantly lower rate of regional disease (IRR: 0.79; 0.71–0.88; p < 0.001), a lower rate of distant disease that did not reach statistical significance (IRR: 0.92; 0.84–1.00; p = 0.052), and no significant difference for localized disease (IRR: 0.98; 0.92–1.04; p = 0.44). Each calendar year of diagnosis was associated with a 6% increase in localized-disease incidence (IRR: 1.06; 1.06–1.07; p < 0.001), whereas the associations for regional (IRR: 1.01; 1.00–1.01; p = 0.050) and distant disease (IRR: 1.01; 1.00–1.01; p = 0.041) were minimal, reaching significance only for distant disease.

**Table 2:** Stage-specific multivariable negative binomial regression models of pancreatic ductal adenocarcinoma incidence in relation to age, sex, race/ethnicity, and year of diagnosis, SEER 21 registries, 2004–2023

Variable	Localized only		Regional only		Distant only	
	IRR (95% CI)	p-value	IRR (95% CI)	p-value	IRR (95% CI)	p-value
Age (per 5-year increase)	1.52 (1.51, 1.53)	< 0.001	1.59 (1.57, 1.60)	< 0.001	1.68 (1.67, 1.69)	< 0.001
Sex						
Male	REF	–	REF	–	REF	–
Female	1.06 (1.01, 1.11)	0.020	0.81 (0.75, 0.88)	< 0.001	0.73 (0.69, 0.78)	< 0.001
Race/ethnicity						
Non-hispanic white	REF	–	REF	–	REF	–
Non-hispanic black	1.31 (1.23, 1.40)	< 0.001	1.10 (0.99, 1.22)	0.083	1.43 (1.30, 1.56)	< 0.001
Non-hispanic asian/PI	0.76 (0.70, 0.81)	< 0.001	0.75 (0.67, 0.84)	< 0.001	0.80 (0.73, 0.88)	< 0.001
Non-hispanic AI/AN	0.90 (0.75, 1.07)	0.22	0.74 (0.61, 0.90)	0.003	0.91 (0.80, 1.03)	0.15
Hispanic	0.98 (0.92, 1.04)	0.44	0.79 (0.71, 0.88)	< 0.001	0.92 (0.84, 1.00)	0.052
Year of diagnosis	1.06 (1.06, 1.07)	< 0.001	1.01 (1.00, 1.01)	0.050	1.01 (1.00, 1.01)	0.041

*AI/AN* American Indian/Alaska Native, *CI* confidence interval, *IRR* incidence rate ratio, *PI* Pacific Islander, *REF* reference category

Separate negative binomial models were fitted within each stage stratum, each adjusted for all other variables in the table

## Discussion

The overall incidence of PDAC appears to be increasing in the United States. The diagnosis of localized PDAC also appears to have increased over time, with a more pronounced rise beginning around 2015 [6, 7, 14]. At the same time, distant-stage disease remained by far the most common presentation, underscoring the persistent challenges in early diagnosis (Figs. 1, 2, 3, 4, 5) [3]. Expressed as a share of all staged diagnoses, localized disease rose from 10.8% of cases in 2004 to 19.1% in 2023, while regional disease fell from 15.7 to 14.4% and distant disease fell from 73.5 to 66.4% (Figure S4), indicating a modest but consistent shift in the stage distribution over the study period.

There are several likely explanations for the observed increase in localized PDAC diagnoses. First, both the frequency and the quality of imaging have increased substantially over time. According to Shiang et al., between 2013 and 2023, the number of CT scans performed in the US increased by 19.2%, and the number of MRIs increased by 21.4% [15]. Second, improvements in cross-sectional imaging, MRI, and endoscopic ultrasound (EUS) have increased the ability to detect smaller pancreatic abnormalities that may have gone undetected previously [7]. Third, surveillance programs for high-risk patients with genetic or familial predisposition have become more common. Increased recognition of hereditary pancreatic cancer syndromes, including BRCA1/2, PALB2, Lynch syndrome, hereditary pancreatitis, and familial pancreatic cancer syndromes, has contributed to broader implementation of targeted surveillance strategies utilizing MRI and EUS in high-risk individuals [16]. This increase in imaging frequency, paired with the ever-improving quality and new targeted surveillance strategies, has led to greater incidental detection of pancreatic lesions.

Emerging biomarker-based approaches in the diagnosis and monitoring of pancreatic lesions are also being explored. Current candidates include protein markers, autoantibodies, circulating tumor DNA, microRNAs, methylated DNA, and exosome-based assays. However, most research in this field has focused on symptomatic patients rather than on asymptomatic surveillance cohorts. In patients with pancreatic cysts, cyst-fluid biomarkers such as CEA, KRAS, and GNAS mutations, as well as alterations in TP53, SMAD4, PIK3CA, PTEN, and AKT1, may help risk-stratify mucinous cysts, but these tests remain adjunctive and require further validation before widespread clinical use [4]. Collectively, these developments may contribute to the earlier diagnosis of PDAC.

Notably, the observed increase in early-stage PDAC does not occur in isolation, as overall PDAC incidence has also risen over time in both sexes and all racial/ethnic groups (Figures S2 and S3). This raises the possibility that the findings observed in this study also reflect a broader increase in PDAC burden nationally. Rahib et al. previously projected that pancreatic cancer may become the second leading cause of cancer-related death in the United States by 2030 [8].

Several established risk factors may contribute to the rising incidence of PDAC. Increasing prevalence of obesity, metabolic diseases, diabetes mellitus, chronic pancreatitis, and aging populations are important contributors to increasing pancreatic cancer incidence worldwide, including in the United States [9, 10].

Racial disparities in PDAC incidence were also observed in this study, with non-Hispanic Black patients demonstrating higher incidence rates compared with White patients. These findings are consistent with prior epidemiologic studies demonstrating significantly higher pancreatic cancer incidence and mortality among Black individuals in the United States, with some studies reporting up to 32% higher pancreatic cancer mortality rates in Black individuals compared with White individuals, although currently recognized risk factors alone did not fully explain this disparity [17]. Differences in smoking prevalence, obesity, diabetes, socioeconomic disparities, delayed diagnosis, differences in healthcare access, and biological and environmental factors all likely play a role, although the contribution of each of these factors remains incompletely understood.

This study has several limitations. This study was a retrospective analysis of SEER data, and the SEER database does not provide information on screening or diagnostic pathways, which limits what we can infer about the underlying causes of these patterns. SEER also lacks data on important individual-level risk factors and clinical characteristics, including comorbid conditions, family history, inherited genetic predisposition, and tobacco use, which precludes adjustment for these factors and limits inferences about the drivers of the observed trends. In addition, a substantial number of cases with unknown stage were excluded from the analytic cohort (Figure S1). Because stage is the central exposure in this analysis, this exclusion is an important limitation: if the completeness of staging information improved over the study period, some of the apparent rise in localized-stage incidence could reflect better stage ascertainment rather than a true shift toward earlier detection. This possibility cannot be excluded with the data available in SEER and should temper the interpretation of the stage-specific trends reported here. Further, changes in staging definitions or reporting over time could have influenced the results. However, the dataset’s size and national scope certainly allow for a broad view of trends over a relatively long period.

## Conclusion

In recent years, the data appear to reflect a shift towards earlier detection of PDAC. Incidence of localized disease has increased, while regional-stage incidence has remained essentially flat and distant-stage disease has shown a comparatively small increase over time. This shift likely reflects advancements in imaging, leading to increased rates of incidental findings, better surveillance programs for high-risk patients, improved techniques such as endoscopic ultrasound for detecting smaller lesions, and perhaps even emerging biomarker-based approaches. Regardless, most patients are still diagnosed with advanced-stage disease. Further research is needed to determine whether the increasing proportion of PDAC being diagnosed with only localized disease reflects real earlier detection, greater diagnostic intensity, an increasing underlying disease burden, or a combination of these factors.

Future prospective multicenter investigations and randomized trials evaluating targeted screening and surveillance strategies will be critical to determine whether these trends translate into meaningful improvements in early detection and survival outcomes in PDAC.

## Supplementary Information

Below is the link to the electronic supplementary material.

Supplementary file1 (DOCX 1478 KB)

## Data Availability

The datasets generated during and/or analyzed during the current study are available in the Surveillance, Epidemiology, and End Results (SEER) Program repository, www.seer.cancer.gov.
