# Supplementary material for: Have we gotten better at diagnosing PDAC before it is too late?: Temporal trends in stage at diagnosis of pancreatic ductal adenocarcinoma in the United States
Source: Cancer Causes Control. 2026 Oct 7;37(10):179. doi: 10.1007/s10552-026-02266-6 (PMC13646018; doi:10.1007/s10552-026-02266-6)
Supplement: Supplementary file 1 [file 10552_2026_2266_MOESM1_ESM.docx]

**Supplementary Material**

**Have we gotten better at diagnosing PDAC before it is too late?: Temporal Trends in Stage at Diagnosis of Pancreatic Ductal Adenocarcinoma in the United States**

Dina Hauptschein MD, Paul Kang MS, MPH, Surabhi Amar MD

**Supplementary Figures**

Figure S1. Cohort construction. Flow diagram showing identification of the analytic cohort of pancreatic ductal adenocarcinoma cases from the SEER 21-registry research data (2000–2023), including the number of rows removed at each exclusion step.

Figure S2. Annual percent change in age-adjusted incidence rates of pancreatic ductal adenocarcinoma (all stages combined), by sex, 2004–2023.

Figure S3. Annual percent change in age-adjusted incidence rates of pancreatic ductal adenocarcinoma (all stages combined), by race/ethnicity, 2004–2023.

Figure S4. Percent distribution of stage at diagnosis among staged pancreatic ductal adenocarcinoma cases, 2004–2023. Localized disease increased from 10.8% of staged diagnoses in 2004 to 19.1% in 2023; regional disease decreased from 15.7% to 14.4%; and distant disease decreased from 73.5% to 66.4%.

**Supplementary Tables**

Table S1. Joinpoint-identified piecewise annual percent change (APC) in age-adjusted incidence rates of pancreatic ductal adenocarcinoma, by race/ethnicity and stage at diagnosis, 2004–2023.

| **Stratum** | **Time period** | **APC (95% CI); p-value** |
| --- | --- | --- |
| **Sex** | **—** | **Trend was log-linear across the full period; see Figure S2.** |
| **Race/ethnicity** |  |  |
| Non-Hispanic White | 2004–2008 | 2.89 (1.71, 6.00); < 0.001 |
| Non-Hispanic White | 2008–2023 | 1.56 (−0.35, 1.76); 0.054 |
| **Stage at diagnosis** |  |  |
| Localized | 2004–2015 | 2.67 (1.29, 3.75); 0.005 |
| Localized | 2015–2019 | 14.7 (10.9, 19.6); < 0.001 |
| Localized | 2019–2023 | 2.15 (−1.55, 4.57); 0.16 |
| Regional | 2004–2008 | 7.74 (4.25, 15.9); < 0.001 |
| Regional | 2008–2014 | 2.54 (−4.89, 4.04); 0.22 |
| Regional | 2014–2023 | −2.96 (−5.82, −0.76); 0.027 |
| Distant | — | Trend was log-linear across the full period; see Figure 1. |

*APC, annual percent change; CI, confidence interval. Strata for which Joinpoint identified no inflection point are shown as log-linear over the full study period.*

Table S2. Joinpoint-identified piecewise annual percent change (APC) in age-adjusted incidence rates of pancreatic ductal adenocarcinoma by stage at diagnosis, stratified by sex and by race/ethnicity, 2004–2023.

| **Cohort** | **Time period** | **APC (95% CI); p-value** |
| --- | --- | --- |
| **Stage by sex** |  |  |
| Male – localized | 2004–2015 | 2.35 (−0.88, 3.97); 0.095 |
| Male – localized | 2015–2018 | 16.9 (8.77, 21.1); 0.010 |
| Male – localized | 2018–2023 | 3.23 (−1.89, 5.57); 0.12 |
| Male – regional | 2004–2014 | 4.21 (3.28, 5.41); < 0.001 |
| Male – regional | 2014–2023 | −3.62 (−4.72, −2.73); < 0.001 |
| Female – localized | 2004–2015 | 2.88 (1.56, 3.84); 0.002 |
| Female – localized | 2015–2019 | 15.1 (11.6, 20.0); < 0.001 |
| Female – localized | 2019–2023 | 2.61 (−0.68, 5.03); 0.081 |
| Female – regional | 2004–2008 | 8.59 (4.10, 17.7); 0.004 |
| Female – regional | 2008–2014 | 1.89 (−4.87, 4.08); 0.27 |
| Female – regional | 2014–2023 | −2.58 (−6.94, 0.13); 0.054 |
| **Stage by race/ethnicity** |  |  |
| NH White – localized | 2004–2015 | 2.73 (1.40, 3.75); 0.005 |
| NH White – localized | 2015–2019 | 14.8 (11.2, 19.8); < 0.001 |
| NH White – localized | 2019–2023 | 1.65 (−1.67, 4.01); 0.19 |
| NH White – regional | 2004–2008 | 8.02 (6.07, 11.5); < 0.001 |
| NH White – regional | 2008–2015 | 2.04 (0.81, 3.01); 0.015 |
| NH White – regional | 2015–2020 | −4.43 (−6.60, 0.76); 0.052 |
| NH White – regional | 2020–2023 | −0.51 (−3.14, 3.00); 0.67 |
| NH Black – localized | 2004–2014 | 0.87 (−7.27, 4.29); 0.69 |
| NH Black – localized | 2014–2019 | 14.1 (7.73, 25.7); 0.028 |
| NH Black – localized | 2019–2023 | 0.82 (−9.45, 6.11); 0.83 |
| NH Black – regional | 2004–2012 | 5.67 (2.04, 20.2); 0.001 |
| NH Black – regional | 2012–2023 | −2.39 (−7.82, −0.44); 0.015 |
| NH Asian/PI – regional | 2004–2013 | 7.30 (3.45, 18.6); < 0.001 |
| NH Asian/PI – regional | 2013–2023 | −2.96 (−7.86, −0.42); 0.028 |
| Hispanic – localized | 2004–2014 | 3.21 (−12.7, 7.73); 0.43 |
| Hispanic – localized | 2014–2023 | 9.21 (6.26, 20.9); 0.028 |
| Hispanic – regional | 2004–2013 | 5.41 (3.18, 10.3); < 0.001 |
| Hispanic – regional | 2013–2023 | −2.16 (−4.56, −0.59); 0.013 |

*APC, annual percent change; CI, confidence interval; NH, Non-Hispanic; PI, Pacific Islander. Only strata for which Joinpoint identified at least one inflection point are shown.*
